# Supplementary figures and images for: Functional genetic variants in complement component 7 confer susceptibility to gastric cancer
Source: PeerJ. 2022 Jan 18;10:e12816. doi: 10.7717/peerj.12816 (PMC8781313; doi:10.7717/peerj.12816)

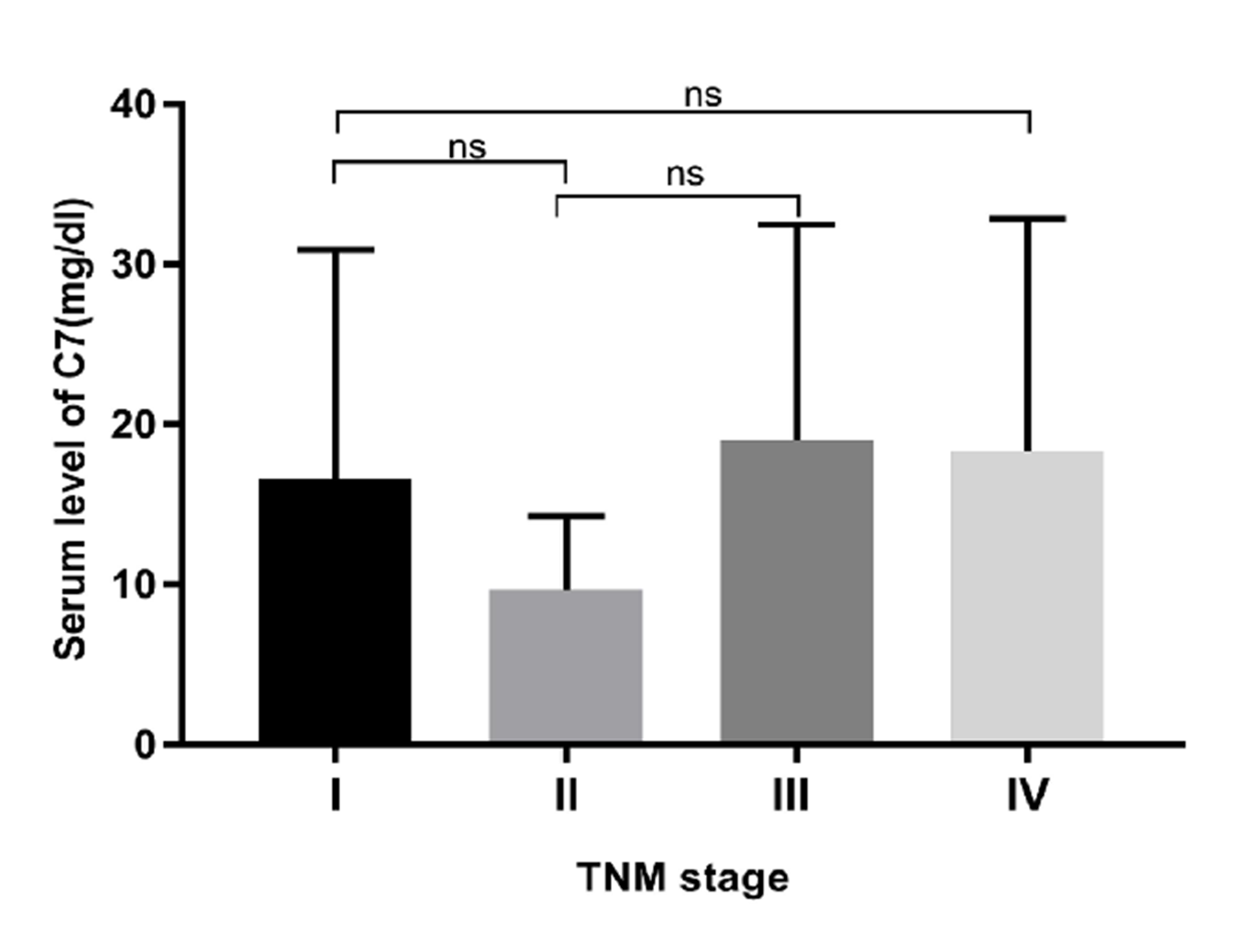

Supplement: Supplemental Information 1 [file peerj-10-12816-s001.png]
